# The use of cIMT as a predictor of postoperative stroke in patients undergoing surgical repair of acute type a aortic dissection

**DOI:** 10.1186/s13019-020-01100-7

**Published:** 2020-04-15

**Authors:** Kai Zhang, Si-Chong Qian, Xu-Dong Pan, Song-Bo Dong, Jun Zheng, Hong Liu, Yue-Li Wang, Li-Zhong Sun

**Affiliations:** 1 Department of Cardiovascular Surgery, Beijing Anzhen Hospital, Capital Medical University, Beijing Institute of Heart, Lung and Blood Vessel Diseases, 2 Anzhen Rd, Beijing, 100029 China; 2 Department of Cardiothoracic Surgery, Chinese Academy of Medical Sciences & Peking Union Medical College, Beijing, China; 3 Department of Echocardiography, Beijing Anzhen Hospital, Capital Medical University, Beijing, China

**Keywords:** Carotid intima–media thickness (cIMT), Type A aortic dissection, Stroke, Risk factor

## Abstract

**Background:**

Acute type A aortic dissection (ATAAD) is a life-threatening condition that requires surgical intervention. Stroke remains an extremely serious adverse outcome that can occur in ATAAD patients undergoing aortic arch repair, leading to higher rates of patient mortality and decreased postoperative quality of life. In the present study, we sought to determine whether carotid intima–media thickness (cIMT) is a reliable predictor of postoperative stroke risk.

**Materials and methods:**

This was a prospective study of 76 patients with ATAAD undergoing aortic arch repair. For all patients, cIMT was determined preoperatively through a Doppler-based method. Incidence of different forms of neurological dysfunction, including temporary neurological dysfunction (TND) and stroke, was monitored in these patients, and the relationship between cIMT and stroke incidence was assessed using a receiver-operating characteristic (ROC) curve. Prognostic variables associated with stroke risk were further identified through univariate and multivariate analyses.

**Results:**

A total of 26/76 (34.2%) patients in the present study suffered from neurological dysfunction, of whom 16 (21.0%) suffered from TND and 10 (13.2%) suffered a stroke. The remaining 50 patients (65.8%) did not suffer from neurological dysfunction. The cIMT values in the stroke, TND, and neurological dysfunction-free patients in this study were 1.12 ± 0.19 (mm), 0.99 ± 0.13 (mm), and 0.87 ± 0.13 (mm), respectively. A total of 4 patients in this cohort died during the study, including 1 in the TND group and 3 in the stroke group. An ROC curve analysis indicated that cIMT could predict stroke with an area under the curve value of 0.844 (95% CI, 0.719–0.969; *p* < 0.001). A multivariate analysis revealed that cIMT > 0.9 mm was independently associated with stroke risk (*p* = 0.018).

**Conclusion:**

We found that cIMT can be used to predict postoperative stroke risk in ATAAD patients undergoing aortic arch repair, with a cIMT > 0.9 mm coinciding with increased stroke risk in these patients.

**Trial registration:**

ChiCTR1900022289. Date of registration 4 April 2019 retrospectively registered.

## Introduction

Acute type A aortic dissection (ATAAD) is a particularly deadly condition, with extremely high rates of intraoperative morbidity and mortality. Surgical treatment of ATAAD patients is primarily focused on saving the lives of these individuals. While associated surgical approaches have improved substantially in recent years, aortic arch repair in ATAAD patients remains a complex procedure with significant associated risk.

A number of technical improvements to aortic arch repair procedures have been described in recent years [1–4], including the use of hypothermic circulatory arrest as a means of improving cerebral protection and the use of selective cerebral perfusion as a mains of safely extending arrest duration. Even with these improvements, however, postoperative stroke remains an extremely serious complication of aortic arch repair surgery that can result in high rates of patient mortality and a significant reduction in postoperative patient quality of life [5–7].

A number of studies in recent years have examined carotid intima–media thickness (cIMT) as a predictor for the risk of cerebral and cardiovascular events in patients [8–10]. Whether cIMT can similarly be used to predict the risk of postoperative stroke in ATAAD patients undergoing aortic arch repair, however, remains to be definitely determined. To that end, in the present study we sought to assess the prognostic relevance of preoperative cIMT as a predictor of postoperative stroke incidence in ATAAD patients undergoing aortic arch surgery.

## Materials and methods

The Ethics Committee of Beijing Anzhen Hospital approved this study. This study was registered in the Chinese Clinical Trials Registry (www.chictr.org.cn, ChiCTR1900022289).

### Patients

This prospective observational study was conducted between December 2016 and June 2017. Patients enrolled in the present study were those ATAAD patients who were at least 18 years of age and who were undergoing total arch replacement (TAR) with frozen elephant trunk (FET) implantation. Exclusion criteria included a history of stroke, aortic surgery, pregnancy, or death during the operation. Patients with coma, altered consciousness, or speech slurring before operations were also excluded. During surgery, both moderate hypothermic circulatory arrest (MHCA) and antegrade selective cerebral perfusion (SCP) were employed in all patients.

**Fig. 1:**
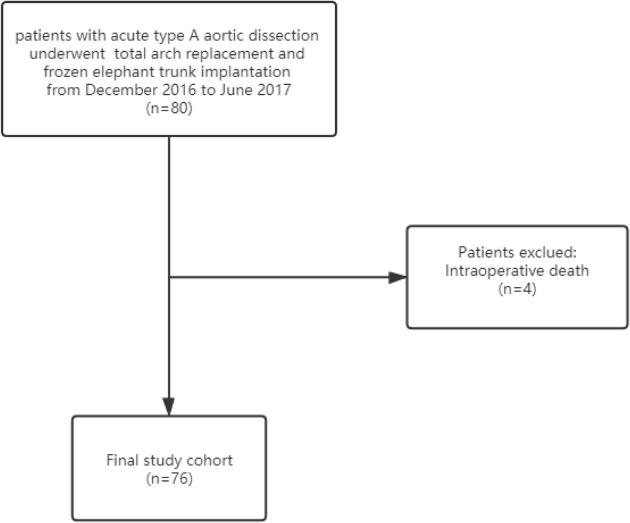
Flow chart diagram

In total, 80 ATAAD patients that underwent TAR with FET were eligible for study enrollment, of whom 4 were excluded due to intraoperative death. The remaining 76 ATAAD patients were enrolled in this study. All operations were performed randomly by four surgery teams. A flow chart of the screening and registration of study participants was given in Fig. 1.

### cIMT measurement

A standard ultrasound procedure with a 9-MHz transducer (GE Vivid E9, Horten, Norway) was used conducted by two highly-experienced sonographers in order to obtain images of the cIMT. Inter-observer variability was determined by the measurement of cIMT three times by each investigator in 10 healthy volunteer participants (*p* = 0.672).

A certified reader examined each image, manually drawing a continuous line at the cIMT interface in these images after which cIMT values were determined. The right and left cIMT measurements over a 1 cm segment of the carotid artery ~ 0.5 cm below the carotid artery bulb not containing any plaques (cIMT ≥1.9 mm) were averaged together to determine cIMT values.

### Definitions

Stroke and temporary neurological dysfunction (TND) were both classified as forms of neurological dysfunction in the present study. Stroke was defined as the development of any several global or focal neurological deficits including altered consciousness or speech slurring as a result of hemorrhagic or ischemic events. Stroke was confirmed using computed tomography (CT) imaging. TND was diagnosed based on the presence of prolonged postoperative confusion, motor weakness, seizures, agitation, or delirium. In TND patients, CT findings were typically normal.

### Surgical technique

All operations were performed through median sternotomy. Briefly, this procedure was performed using right axillary artery cannulation for cardiopulmonary bypass (CPB) and SCP [5–10 mL/(kg•min)] under MHCA. Associated operations, such as mitral valve and aortic root replacement, were performed during cooling. The TAR with FET surgical technique has been previously described [2, 11]. The procedure involved the implantation of a FET (MicroPort Medical Company Limited, Shanghai, China) in the descending aorta, followed by TAR with a four-branched prosthetic graft (Maquet Cardiovascular, Wayne, NJ). Distal reperfusion was initiated upon the completion of descending aortic anastomosis. The left carotid artery was initially reconstructed to achieve bilateral perfusion, the ascending aorta was then treated to prevent coronary ischaemia, and the innominate and subclavian arteries were treated last.

### Statistical analysis

Continuous and categorical data were given as mean ± standard deviation (SD) or percentage, and were compared via t-tests or Kruskal-Wallis tests and chi-squared tests or Fisher’s exact tests, respectively. Logistic regression analyses were used to identify variables associated with postoperative stroke risk, with a multivariate analysis used to assess the link between patient stroke risk cIMT > 0.9 mm [12]. Covariates were adjusted based upon previous reports [13]. Briefly, when the addition of a variable resulted in a change in the odds ratio of ≥10%, adjustments were made. Covariates in this model included male, body mass index (BMI), preoperative white blood cell count, coronary artery involvement, circulatory arrest time, hydropericardium, and Marfan syndrome. This cIMT cutoff value used to predict postoperative stroke risk was determined based upon a receiver-operating characteristic (ROC) curve. Youden’s index (J = Sensitivity + Specificity - 1) was used to determine the optimal cIMT cut-off value for this curve. All tests were 2-sided, with *p* < 0.05 as the significance threshold. R and Empower Stats (http://www.empowerstats.com, X&Y Solutions, Inc., MA, USA) was used for all statistical testing.

## Results

A total of 76 patients were included in this analysis, of whom 50 did not suffer from any neurological dysfunction (65.8%), 16 suffered from TND (21.1%), and 10 suffered from stroke (13.2%). Table 1 shows both the baseline and operative characteristics for these patients. Patients in the stroke, TND, and neurological dysfunction-free (NDF) groups exhibited cIMT values of 1.12 ± 0.19 (mm), 0.99 ± 0.13 (mm), and 0.87 ± 0.13 (mm), respectively (*p* < 0.001). Just 15/50 (30.0%) of patients in the NDF group exhibited a cIMT value > 0.9 mm, whereas 12/16 (75.0%) and 8/10 (80%) patients in the TND and stroke groups, respectively, had a cIMT value > 0.9 mm (*p* < 0.001). All other measured variables were comparable among these three patient groups.

**Table 1:** Demographic characteristics, preoperative and intraoperative data of patients

Variable	Total (***n*** = 76)	NDF (***n*** = 50)	TND (***n*** = 16)	Stroke (***n*** = 10)	***P*** value
Male	61 (80.3)	40 (80.0)	15 (97.8)	6 (60.0)	0.109
Age (year)	45.8 ± 9.9	45.3 ± 10.6	48.1 ± 9.0	47.0 ± 8.4	0.799
BMI (kg/m^2^)	27.4 ± 4.6	27.4 ± 4.2	27.8 ± 5.8	26.8 ± 4.9	0.863
Hypertension	60 (78.9)	41 (82.0)	12 (75.0)	7 (70.0)	0.634
Diabetes mellitus	3 (3.9)	3 (6.0)	0	0	0.444
Marfan syndrome	1 (1.3)	0	1 (6.3)	0	0.150
Smoking history	33 (43.4)	21 (42.0)	9 (56.3)	3 (30.0)	0.397
LVEF (%)	62.2 ± 4.9	62.3 ± 4.5	60.7 ± 6.5	63.9 ± 3.6	0.258
White blood cell count (10^9^/l)	12.0 ± 3.0	11.5 ± 3.0	12.99 ± 3.6	12.8 ± 1.8	0.155
Serum creatinine (umol/l)	84.7 ± 31.6	78.1 ± 20.4	105.9 ± 51.6	84.4 ± 24.3	0.142
Primary entry tear					
Unknown	14 (18.4)	7 (14.0)	4 (25.0)	3 (30.0)	0.367
Ascending aorta	57 (75.0)	38 (76.0)	12 (75.0)	7 (70.0)	0.923
Aortic arch	5 (6.6)	5 (10.0)	0	0	0.993
Coronary artery involvement	20 (26.3)	17 (34.0)	1 (6.3)	2 (20.0)	0.628
Aortic root repair					0.606
Ascending aorta replacement	50 (65.8)	31 (62.0)	12 (75.0)	7 (70.0)	
Bentall procedure	26 (34.2)	19 (38.0)	4 (25.0)	3 (30.0)	
Lowest nasopharyngeal temperature (°C)	23.5 ± 0.8	23.5 ± 0.8	23.7 ± 0.7	23.7 ± 0.5	0.412
CPB time (min)	205.8 ± 43.2	199.5 ± 41.6	223.9 ± 45.3	207.8 ± 43.6	0.143
Cross-clamp time (min)	114.2 ± 30.5	109.9 ± 30.5	129.5 ± 29.8	111.0 ± 26.5	0.724
Circulatory arrest time (min)	22.9 ± 7.5	22.0 ± 7.5	26.4 ± 7.6	22.2 ± 6.1	0.737
Intraoperative transfusion of PRBCs	44 (57.9)	27 (54.0)	12 (75.0)	5 (50.0)	0.589
Intraoperative transfusion of FFP	38 (50.0)	24 (48.0)	10 (62.5)	4 (40.0)	0.500
Intraoperative transfusion of platelets	5 (6.9)	3 (6.0)	2 (12.5)	0	0.993
cIMT (mm)	0.93 ± 0.16	0.87 ± 0.13	0.99 ± 0.13	1.12 ± 0.19	< 0.001
cIMT (mm) > 0.9	35 (46.5)	15 (30.0)	12 (75.0)	8 (80.0)	< 0.001

Continuous data are presented as the mean ± standard deviation and categorical data as number (percentage)

*Abbreviations*: *NDF* neurological dysfunction-free, *TND* temporary neurological dysfunction, *BMI* body mass index, *LVEF* left ventricular ejection fraction, *CABG* coronary artery bypass grafting, *CPB* cardiopulmonary bypass, *PRBCs* packed red blood cells, *FFP* fresh-frozen plasma, *cIMT* carotid intima–media thickness

### Operative morbidity and mortality

Table 2 showed the postoperative complications of these patients. Overall patient mortality in the present study was 5.3% (4 of 76), with all deceased patients having suffered from some form of neurological dysfunction (1 TND, 3 stroke) (*p* < 0.01). A total of 8 patients (10.5%) underwent postoperative continuous renal replacement therapy, including 3 in the TND group, and 5 in the stroke group (*p* < 0.01). Patients that suffered from postoperative neurological dysfunction also had higher rates of tracheal reintubation (3/10, 30.0%; 4 /16, 25.0%; vs 1/50, 2.0%; *p* = 0.003). A total of 2 (20.0%), 1 (6.3%), and 4 (8.0%, *p* = 0.439) patients in the stroke, TND, and NDF groups suffered from postoperative paraplegia. Patients that suffered from stroke or TND also had higher rates of requiring tracheotomy (20% and 12.5%, respectively, vs 2%; *p* = 0.037) and reexploration for bleeding (20% and 12.5%, respectively vs 2%; *p* = 0.037).

**Table 2:** Postoperative complications of patients

Variable	Total (***n*** = 76)	NDF (***n*** = 50)	TND (***n*** = 16)	Stroke (***n*** = 10)	***P*** value
Paraplegia	7 (9.2)	4 (8.0)	1 (6.3)	2 (20.0)	0.439
Continuous renal replacement therapy	8 (10.5)	0	5 (31.3)	3 (30.0)	< 0.001
Tracheal reintubation	8 (10.5)	1 (2.0)	4 (25.0)	3 (30.0)	0.003
Tracheotomy	5 (6.6)	1 (2.0)	2 (12.5)	2 (20.0)	0.037
Reexploration for bleeding	5 (6.6)	1 (2.0)	2 (12.5)	2 (20.0)	0.037
Mortality	4 (5.3)	0	1 (6.3)	3 (30.0)	< 0.001

Continuous data are presented as the mean ± standard deviation and categorical data as number (percentage)

*Abbreviations*: *NDF* neurological dysfunction-free, *TND* temporary neurological dysfunction

### Univariate and multivariate analysis

A univariate analysis revealed that of the tested variables, only cIMT > 0.9 mm was associated with increased stroke incidence (OR, 5.78; 95% CI, 1.14–29.35; *p* = 0.034; Table 3). A multivariate analysis was then performed to confirm this finding, incorporating covariates including male, BMI, preoperative white blood cell count, coronary artery involvement, circulatory arrest time, hydropericardium, and Marfan syndrome (Table 4). Following adjustment for these covariates, cIMT > 0.9 mm was still associated with increased stroke risk (OR 5.78, 95% CI 1.14–29.35; *p* = 0.018).

**Table 3:** Univariate analysis of risk factors associated with stroke

Variables	No-stroke (***n*** = 66)	Stroke (***n*** = 10)	OR(95%CI)	***P*** value
Male	55 (83.3)	6 (60.0)	0.30 (0.07–1.24)	0.097
Age (year)	45.6 ± 10.2	47.0 ± 8.4	1.01 (0.95–1.09)	0.684
BMI (kg/m^2^)	27.5 ± 4.6	26.8 ± 4.9	0.96 (0.82–1.13)	0.638
Hypertension	53 (80.3)	7 (70.0)	0.57 (0.13–2.52)	0.461
Diabetes mellitus	3 (4.5)	0	a	0.995
Hyperlipidemia	5 (7.6)	1 (10.0)	1.36 (0.14–12.97)	0.792
Marfan syndrome	1 (1.5)	0	a	0.995
Smoking history	30 (45.5)	3 (30.0)	0.51 (0.12–2.16)	0.364
Hydropericardium	11 (16.7)	2 (20.0)	1.25 (0.23–6.70)	0.795
LVEF (%)	61.9 ± 5.1	63.9 ± 3.6	1.09 (0.94–1.26)	0.241
White blood cell count (10^9^/l)	11.9 ± 3.2	12.8 ± 1.8	1.11 (0.89–1.38)	0.346
Serum creatinine (umol/l)	84.7 ± 32.7	84.4 ± 24.3	1.00 (0.98–1.02)	0.973
Primary entry tear				
Unknown	11 (16.7)	3 (30.0)	1.0	
Ascending aorta	50 (75.8)	7 (70.0)	0.51 (0.11–2.30)	0.384
Aortic arch	5 (7.6)	0	a	0.993
Coronary artery involvement	18 (27.3)	2 (20.0)	0.67 (0.13–3.44)	0.628
Aortic root repair				
Ascending aorta replacement	43 (65.2)	7 (70.0)	1.0	
Bentall procedure	23 (34.9)	3 (30.0)	1.09 (0.53–2.24)	0.806
Lowest nasopharyngeal temperature (°C)	23.5 ± 0.8	23.7 ± 0.5	1.47 (0.61–3.56)	0.394
CPB time (min)	205.4 ± 43.5	207.8 ± 43.6	1.00 (0.99–1.02)	0.871
Cross-clamp time (min)	114.6 ± 31.3	111.0 ± 26.5	1.00 (0.97–1.02)	0.724
Circulatory arrest time (min)	23.1 ± 7.7	22.2 ± 6.1	0.98 (0.89–1.08)	0.737
Intraoperative transfusion of PRBCs	39 (59.1)	5 (50.0)	0.69 (0.18–2.63)	0.589
Intraoperative transfusion of FFP	34 (51.5)	4 (40.0)	0.63 (0.16–2.43)	0.500
Intraoperative transfusion of platelets	5 (7.6)	0	a	0.993
cIMT (mm) > 0.9	27 (40.9)	8 (80.0)	5.78 (1.14–29.35)	0.034

Continuous data are presented as the mean ± standard deviation and categorical data as number (percentage)

*Abbreviations*: *OR* odd ratio, *95% CI* 95% confidence interval, *BMI* body mass index, *LVEF* left ventricular ejection fraction, *CABG* coronary artery bypass grafting, *CPB* cardiopulmonary bypass, *PRBCs* packed red blood cells, *FFP* fresh-frozen plasma, *cIMT* carotid intima–media thickness

^a^The result failed because of the small sample size

**Table 4:** Multivariable analysis to assess the independent impact of cIMT group on stroke in patients with ATAAD

Variable	Model I OR (95% CI)	*P*-value	Model II OR (95% CI)	*P*-value
cIMT (mm) > 0.9	5.78 (1.14–29.35)	0.034	9.53 (1.47–61.72)	0.018

*Abbreviations*: *cIMT* carotid intima–media thickness, *ATAAD* acute type A aortic dissection, *OR* odd ratio, *95% CI* 95% confidence interval

Model I: unadjusted

Model II: adjusted for male, BMI, Marfan syndrome, hydropericardium, preoperative white blood cell count, coronary artery involvement, circulatory arrest time

### Area under the ROC curve analysis

**Fig. 2:**
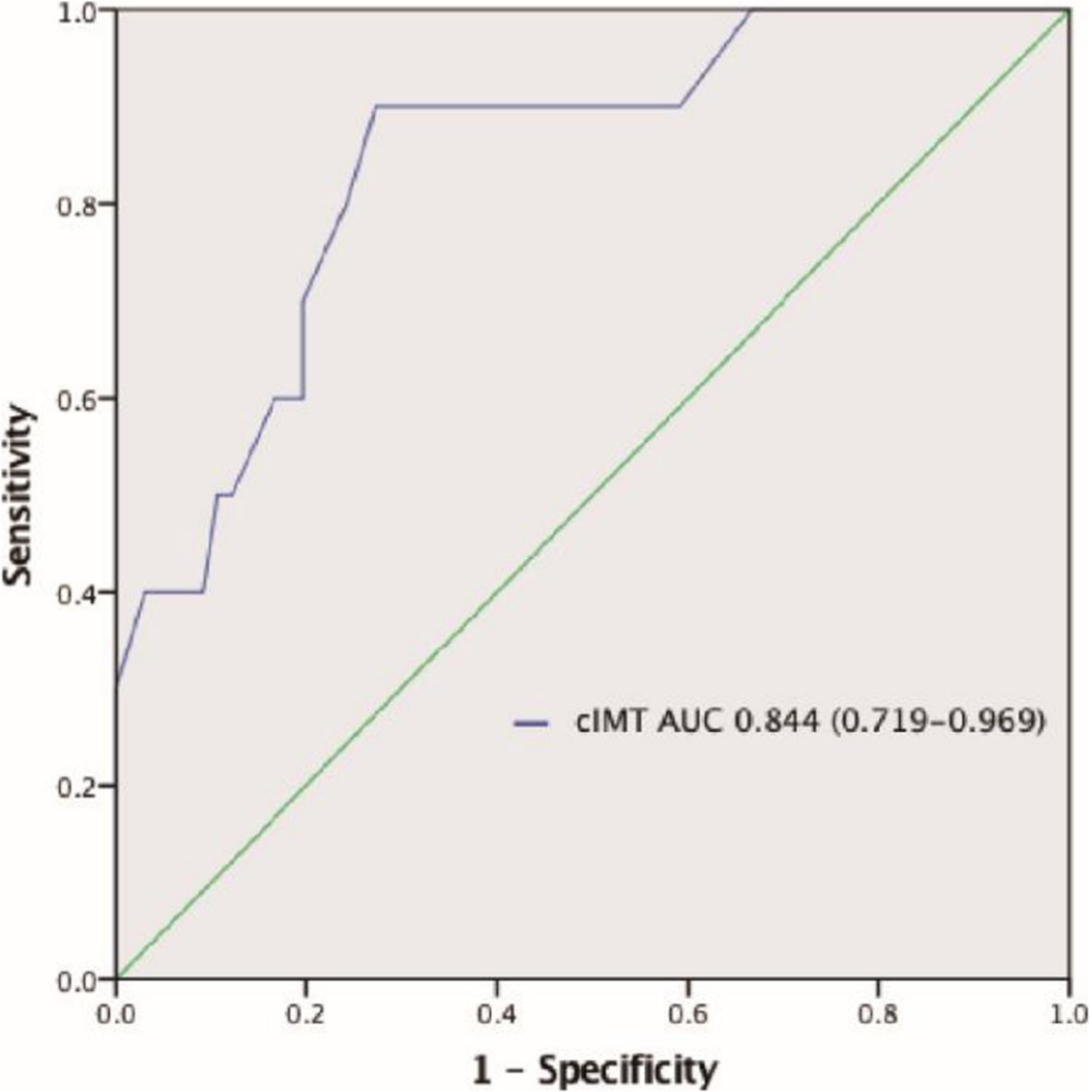
Receiver-operating characteristic of cIMT before the operation. Abbreviations: AUC, areas under receiver-operating characteristic curve; cIMT, carotid intima–media thickness

The area under the ROC curve for cIMT values in the present study was found to be 0.844 (95% CI, 0.719–0.969; *p* < 0.001; Fig. 2). This analysis confirmed that cIMT offered significant value as a predictor of stroke risk (*p* < 0.001), with sensitivity and specificity values of 90.0% and 72.7%, respectively. The cIMT cutoff value for diagnosing stroke was 0.627.

## Discussion

Our study revealed that the noninvasive measurement of cIMT using carotid ultrasonography can predict the risk of postoperative stroke in ATAAD patients undergoing aortic arch repair with high sensitivity and specificity (90.0% and 72.7%, respectively). Specifically, we determined that cIMT > 0.9 mm was independently associated with increased risk of postoperative stroke in these individuals.

While there have been significant improvements in ATAAD patient outcomes following surgical aortic arch repair, the rates of morbidity and mortality associated with the procedure remain high with cerebral complications affecting from 7% to 35% of patients [14, 15]. The most recent international registry of aortic dissection study of ATAAD [16] reported an approximate 10% rate of cerebral complications, with the latest Society of Thoracic Surgeons Adult Cardiac Surgery Database study [1] reporting a 13% risk of postoperative stroke following surgical repair of ATAAD (945/7353 patients across 772 centers). These findings were consistent with the results of our smaller scale study (10/76, 13.2%).

Many recent studies have utilized cIMT as a key predictor of cardiovascular risk [8–10], but to date no studies have focused on this parameter as a predictor of postoperative stroke risk in patients following aortic arch surgery. In the present study, we therefore examined the ability of cIMT to predict such postoperative stroke risk in ATAAD patients given that cIMT has been previously shown in other contexts to independently predict postoperative stroke risk even in light of more traditional predictors of cardiovascular risk [17–20]. Recent guidelines published by the American College of Cardiology Foundation–American Heart Association guidelines also highlight cIMT as having a level IIa recommendation for cardiovascular risk evaluation [21].

At present the clinical parameters that are associated with postoperative stroke risk following aortic arch repair remain uncertain, although previous studies have found that postoperative stroke are among the primary drivers of operative mortality [5–7, 22]. Some studies have also found that deep hypothermic circulatory arrest (DHCA) time [23] and history of stroke [24] were all associated with postoperative stroke risk, but other studies have failed to replicate these findings with multivariate analyses suggesting that DHCA time was not predictive of postoperative stroke incidence [24, 25]. In our study, CPB time, cross-clamp time, and circulatory arrest time, all of which are usually deemed to be associated with cerebral ischemia risk, were found not to be significantly associated with stroke incidence in a univariate analysis. We believe there may be three reasons for this finding: First, prolonged operation time is known to lead to higher rates of adverse outcomes, but our experienced surgeons were able to maintain relatively low durations of CPB, cross-clamp, and circulatory arrest in this study. In addition, SCP significantly extended the safe arch intervention time and increased brain tissue tolerance in these patients. Second, when we divided the patients into three groups based on neurological outcomes, it was clear that patients in the TND group experienced longer CPB, cross-clamp, and circulatory arrest durations, which may have resulted in transient ischemia but not substantial brain damage in these patients. Third, this study had a relatively small sample size and thus limited statistical power, and as such these variables were still incorporated into the final multivariate logistic regression analyses in order to ensure that the results are as accurate as possible. In the present study, of analyzed variables only a cIMT value > 0.9 mm was found to be independently associated with stroke risk. This parameter serves as a valuable index for quantifying vascular abnormalities.

In patients with ATAAD, it is imperative that surgery be conducted as quickly as possible as mortality rates rise in a time-dependent manner, and as such only essential examinations should be performed before surgery is initiated. Owing to its ease of measurement and ability to predict stroke risk, cIMT values thus represent a feasible and potentially valuable tool for stratifying ATAAD patients based upon risk. The ability to determine which patients are at a high-risk of postoperative stroke may have important clinical implications, as more attention can then be paid to these high-risk individuals. When symptoms are evident in these patients, cranial CT examination should be performed immediately, and treatment should be conducted as early as possible to ensure an optimal prognosis.

### Study limitations

This study has a number of limitations. For one, this was a single-center study with a relatively small number of patients. In addition, there is potential for selection bias as we focused specifically on ATAAD patients that underwent TAR with FET, whereas other studies may not have selected patients in this manner leading to differences in study outcomes. Third, We did not have adequate cranial CT to confirm all of our enrolled patients did not suffer from stoke before operation. Fourth, we did not assess long-term morbidity or mortality in patients that suffered from stroke in the present study. Despite these limitations, our data still suggests that cIMT values may be of benefit for detecting stroke risk, thereby offering an opportunity to improve outcomes in ATAAD patients undergoing aortic arch repair.

## Conclusions

Our analyses clearly indicate that cIMT can be used as a reliable predictor of postoperative stroke risk in ATAAD patients scheduled to undergo aortic arch repair. We found a cIMT value > 0.9 mm to be associated with increased stroke risk in these patients with good specificity and sensitivity.

## Data Availability

The datasets used or analyzed during the current study are available from the corresponding author on reasonable request.
